# Low-temperature culture enhances production of flavivirus virus-like particles in mammalian cells

**DOI:** 10.1007/s00253-024-13064-y

**Published:** 2024-02-28

**Authors:** Yi-Chin Fan, Jo-Mei Chen, Yi-Ying Chen, Wei-Li Hsu, Gwong-Jen Chang, Shyan-Song Chiou

**Affiliations:** 1 Graduate Institute of Microbiology and Public Health, National Chung Hsing University, Taichung, 402 Taiwan; 2 Institute of Epidemiology and Preventive Medicine, College of Public Health, National Taiwan University, Taipei, 10617 Taiwan; 3 Arboviral Diseases Branch, Centers for Disease Control and Prevention, Fort Collins, CO 80521 USA

**Keywords:** Flavivirus, Virus-like particles, Mammalian cells, Cultivation condition

## Abstract

**Abstract:**

Flavivirus virus-like particles (VLPs) exhibit a striking structural resemblance to viral particles, making them highly adaptable for various applications, including vaccines and diagnostics. Consequently, increasing VLPs production is important and can be achieved by optimizing expression plasmids and cell culture conditions. While attempting to express genotype III (GIII) Japanese encephalitis virus (JEV) VLPs containing the G104H mutation in the envelope (E) protein, we failed to generate VLPs in COS-1 cells. However, VLPs production was restored by cultivating plasmid-transfected cells at a lower temperature, specifically 28 °C. Furthermore, we observed that the enhancement in JEV VLPs production was independent of amino acid mutations in the E protein. The optimal condition for JEV VLPs production in plasmid-transfected COS-1 cells consisted of an initial culture at 37 °C for 6 h, followed by a shift to 28 °C (37/28 °C) for cultivation. Under 37/28 °C cultivation conditions, flavivirus VLPs production significantly increased in various mammalian cell lines regardless of whether its expression was transiently transfected or clonally selected cells. Remarkably, clonally selected cell lines expressing flavivirus VLPs consistently achieved yields exceeding 1 μg/ml. Binding affinity analyses using monoclonal antibodies revealed similar binding patterns for VLPs of genotype I (GI) JEV, GIII JEV, West Nile virus (WNV), and dengue virus serotype 2 (DENV-2) produced under both 37 °C or 37/28 °C cultivation conditions. In summary, our study demonstrated that the production of flavivirus VLPs can be significantly improved under 37/28 °C cultivation conditions without affecting the conformational structure of the E protein.

**Keypoints:**

• *Low-temperature culture (37/28 °C) enhances production of flavivirus VLPs.*

• *Flavivirus VLPs consistently achieved yields exceeding 1 μg/ml.*

• *37/28 °C cultivation did not alter the structure of flavivirus VLPs.*

**Supplementary Information:**

The online version contains supplementary material available at 10.1007/s00253-024-13064-y.

## Introduction

The spread of flaviviruses presents a significant global health threat, exemplified by the resurgence of DENV after 1980 (Guzman et al. 2010), the introduction of the WNV to the United States in 1999 (Asnis et al. 2000), and the Zika virus threat during the 2016 Brazilian Olympics (Weaver et al. 2016). Many of these viruses lack vaccines for effective outbreak control (Crunkhorn 2021), and the serological testing of these viruses is complicated due to their antibody cross-reactivity elicited by the antigenic similarities among them (Tsai et al. 2020). Established systems for constructing and expressing flavivirus VLPs offer promising solutions to address these challenges (Cuevas-Juarez et al. 2021).

Flaviviruses are positive-sense RNA viruses with a single open reading frame. The initial one-third of viral RNA encodes three structural proteins, while the remaining two-thirds encode non-structural proteins (Chambers et al. 1990). Since the late 1990s, systems for expressing flavivirus VLPs have been established by the transfection of mammalian cell lines with plasmids containing premembrane (prM) and E genes (Chang et al. 2000; Cuevas-Juarez et al. 2021; Shang et al. 2012). This process permits the unique property of prM-E protein to self-assemble into VLPs closely resembling the authentic viral structure. Due to their structural and antigenic similarity to viral particles, these VLPs have been explored and considered for non-infectious vaccine development. Although commercial flavivirus VLPs vaccines are unavailable, animal experiments have demonstrated robust protective effects elicited by VLP vaccine candidates (Cuevas-Juarez et al. 2021). Moreover, owing to their non-infectious nature, VLPs are frequently employed as diagnostic antigens for serological testing. Their use eliminates the need for various inactivation processes that potentially alter viral protein structure, resulting in improved accuracy in antibody diagnostics compared to inactivated viral particles (Chiou et al. 2008; Holmes et al. 2005; Mali and Bondre 2022).

Flavivirus VLPs offer numerous potential applications, making it critically important to enhance VLPs production and yield. Generally, two approaches have been employed to increase flavivirus VLPs expression. The first involves constructing plasmids with improved expression capabilities, which includes methods such as codon optimization, the use of appropriate promoters, signal sequences, and poly(A) tails (Chang et al. 2000). The second method focuses on optimizing the protein expression process, encompassing strategies like selecting suitable cell lines (Yamaji et al. 2013), increasing cell culture density, generating stably expressing cell lines, and adjusting cell culture media composition (Kojima et al. 2003). Effectively employing these methods enhances the production yield of flavivirus VLPs, opening possibilities for widespread utilization and commercialization.

In one previous study, we encountered difficulties in producing JEV VLPs with the G104H mutation in the E protein when using COS-1 cells as the expression cell line. However, we successfully resolved this issue by implementing low-temperature cell culture techniques (Chiou et al. 2008). In this study, we applied this temperature switch approach to produce various flavivirus VLPs, resulting in a substantial increase in production yield in mammalian cells either by transient expression or using stably selected cell lines.

## Materials and methods

### Virus-like particles (VLPs) expression plasmids

The wild-type (WT) Japanese encephalitis virus (JEV) VLPs expression plasmid was previously created using the genotype III (GIII) SA14 strain sequence (Chiou et al. 2008). This plasmid, designated as pVAX-JEi, contains various elements including the human cytomegalovirus (CMV) early gene promoter, a Kozak sequence, the signal sequence of JEV capsid (C) gene, the complete JEV premembrane/membrane (prM/M) and E gene region, an intron, a bovine growth hormone poly(A) signal [BGH(A)], and a kanamycin resistance gene. To construct VLPs expression plasmids for other WT flaviviruses, a similar approach was taken by starting with the pVAX-JEi plasmid and replacing the prM and E gene region (amino-terminal 80%) with that of the target virus (Purdy and Chang 2005). This process was applied to GI JEV (Fan et al. 2018), West Nile virus (WNV) (Roberson et al. 2007), St. Louis encephalitis virus (SLEV) (Trainor et al. 2007; Roberson et al. 2007), and Dengue virus serotypes 1 to 4 (DENV1-4) (Crill and Chang 2004; Purdy and Chang 2005). The viral genes of pVAX flavivirus VLPs expression plasmids were cleavaged with KpnI and NotI restriction enzymes and subcloned into pCDNA3.1 vector, which contains neomycin resistant gene for stable cell lines selection using Geneticin (G418). Site-specific mutations were introduced into the JEV E gene using a Quick Change multisite-directed mutagenesis kit (Stratagene, La Jolla, Calif.), with pVAX-JEi (GIII and GI) serving as the DNA template, following the manufacturer's recommended protocols.

### WT and mutant VLPs expression

COS-1, Vero, CHO-K1, and 293 T cells were cultured at 37 °C with 5% CO_2_ in Dulbecco's Modified Eagle's Minimal Essential Medium (DMEM; Gibco, Grand Island, NY) supplemented with 5% heat-inactivated fetal bovine serum (HyClone Laboratories, Inc., Logan, UT). For transformation, a cell suspension was electroporated with 20 µg of plasmid DNA using a cuvette with a 0.4-cm electrode gap, employing a Bio-Rad Gene Pulser II (Bio-Rad Laboratories, Hercules, Calif.) set at 250 V and 975 µF. The transformed cells were seeded into 75-cm^2^ culture flasks containing 50 ml of growth medium. Subsequently, they were recovered for 6 h at either 24 °C, 28 °C, or 37 °C, after which they were continually maintained at the designated temperature (24 °C, 28 °C, or 37 °C). The tissue culture medium was harvested, replaced with fresh medium with 2% FBS, clarified by centrifugation at 10,000 rpm for 30 min at 4 °C, utilizing a Sorval F-16/250 rotor (Beckman Coulter), and stored at 4 °C for further analysis.

### Antibodies

To titrate and standardize VLPs antigens, we utilized rabbit anti-flavivirus (JEV, WNV, SLEV, and DENV1-4) polyclonal antibodies and anti-flavivirus (JEV, WNV, SLEV, and DENV1-4) murine hyperimmune ascitic fluid (MHIAF). In the process of antigen characterization, we selected monoclonal antibodies (MAbs) that exhibited cross-reactivity within the flavivirus group, cross-reactivity within the serocomplex, and specificity to individual virus types, as outlined in Table S1.

### Antigen-capture enzyme-linked immunosorbent assay (Ag-ELISA)

The titration and standardization of flavivirus VLPs antigens were conducted using Ag-ELISA, following established procedures (Chiou et al. 2008). In brief, 96-well plates were coated with rabbit anti-flavivirus polyclonal antibodies (Table S1) at 37 °C for 1 h and subsequently blocked with StartBlock blocking buffer (Pierce, Rockford, Ill.). Next, VLPs or negative COS-1 cell antigens were added to the wells and incubated at 4 °C overnight. The captured antigens were then recognized by serially diluted mouse anti-flavivirus murine hyperimmune ascitic fluid (MHIAF) (Table S1) at 37 °C for 1 h. Bound mouse anti-flavivirus antibodies were detected using peroxidase-conjugated goat anti-mouse IgG (H + L) (Jackson ImmunoResearch, West Grove, PA) at 37 °C for 1 h. Unbound antibodies were removed by washing with 1X PBST. Subsequently, TMB substrate (Neogen Corp., Lexington, KY) was added to the wells, and it reacted with peroxidase for 10 min. The reaction was halted by adding 2N H_2_SO_4_. The values of OD_450_ were measured and recorded. The ELISA endpoint titer of the VLP antigens was determined as the reciprocal dilution at which the OD_450_ ratio of the sample to negative antigens (P/N ratio) equaled 2, using GraphPad version 5.01.

To measure the absolute quantification of VLPs, we calculated antigen levels based on absorbance values derived from both the sample and a reference standard, expressing them as the E protein amount in nanograms per milliliter. The reference standard was created using purified and concentrated VLPs obtained from the culture fluids of COS-1 cells transfected with the GIII JEV VLPs expressing plasmid. The E protein amount within the standard VLPs preparation was estimated by comparing it to BSA samples analyzed on Coomassie brilliant blue-stained gels.

To assess MAb reactivity with flavivirus VLPs in Ag-ELISA, a panel of MAbs (Table S1) was employed. The secreted VLPs antigens were standardized by selecting the antigen concentration that yielded an OD_450_ value of approximately 1.0 when using polyclonal anti-flavivirus MHIAF.

### Indirect immunofluorescence assay (IFA)

Transfected COS-1 cells were fixed using 4% paraformaldehyde-PBS and triton X-100, and VLP antigens within the cells were identified using mouse anti-JEV MHIAF. Subsequently, the FITC-conjugated goat anti-mouse IgG antibodies (KPL, Gaithersburg, MD) were added, and cell cytoplasm was stained with Evan blue (Invitrogen). The presence of JEV antigens and cell cytoplasm was visualized and recorded using an OLYMPUS CKX41 microscope.

### Statistical analysis

Statistical analysis was conducted using GraphPad Prism v5.01. A Student's two-tailed t-test was employed to compare two sets of data. *P* < 0.05 indicated a significant difference in the comparison between the two groups.

## Results

### Low-temperature cultivation enhances the production of various JEV VLPs in COS-1 cell lines

**Fig. 1:**
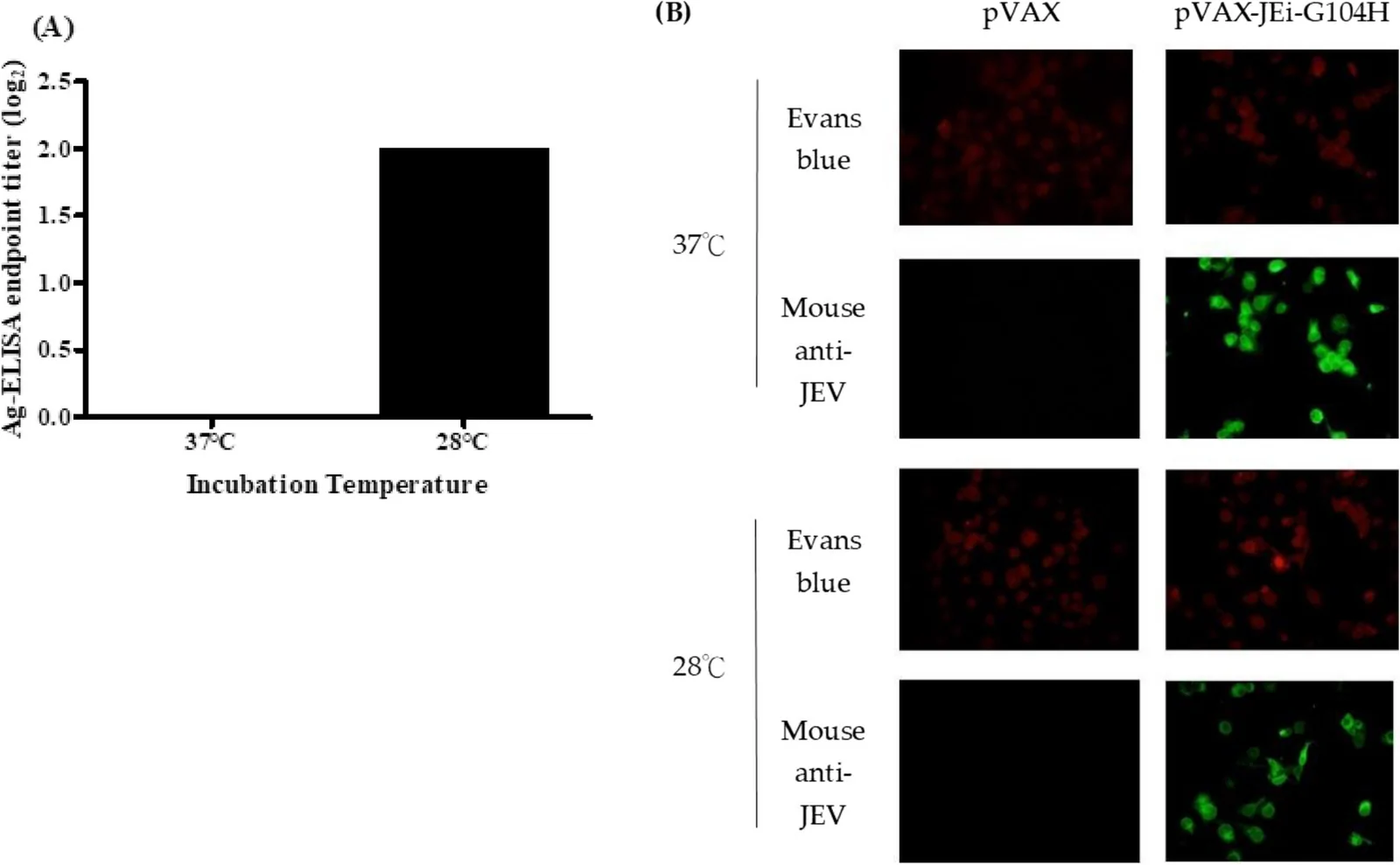
Expression of JEV VLPs in COS-1 cells transfected with G104H-mutated JEV plasmid and incubated at 37℃ or 28℃. The extracellular VLP in cultured supernatant was collected and measured by Ag-ELISA (**A**), and intracellular VLP expression was detected by IFA with anti-JEV MHIAF and cells stained with Evans blue (**B**). These experiments were conducted once

In our previous research, we successfully constructed and expressed GIII JEV VLPs. However, when investigating cross-reactive epitopes, we encountered a problem related to the G104H mutation in the fusion loop of the JEV E protein. This mutation completely hindered the production of JEV VLPs in transfected COS-1 cells cultured at 37 °C (Chiou et al. 2008). Earlier literature had reported similar challenges with amino acid mutations in the Semliki Forest virus (SFV) E1 protein, which could be overcome by culturing infected mammalian Vero cells at 28 °C (Duffus et al. 1995). We, therefore, transfected COS-1 cells with the GIII JEV VLPs expression plasmids containing the G104H mutation and cultured them at 28 °C. Subsequent collection of cell culture supernatants yielded positive results in Ag-ELISA, with titers reaching 1:4, but not samples collected from cells cultured at 37 °C (Fig. 1A and Table 1). Surprisingly, further immunofluorescence assays (IFA) revealed positive signals in transfected cells regardless of their culture temperature at 37 °C or 28 °C (Fig. 1B). These results suggested that the G104H mutation does not affect protein expression but higher temperature at 37 °C may hinder the assembly or release of GIII JEV VLPs. This inhibition can potentially be overcome by reducing the culturing temperature to 28 °C.

**Table 1:** The production of various JEV VLPs in plasmid-transfected COS-1 cells cultured at 37℃ and 28℃

JEV VLP Plasmids	Ag-ELISA (end-point titer)^a^		Fold increased
	37℃	28℃	
GIII WT (pVAX)	1:8	1:64	8
GIII WT (pCDNA3.1)	1:4	1:32	8
GIII W101G (pVAX)	1:16	1:128	8
GIII G104H (pVAX)	-^b^	1:4	≧4
GI WT (pVAX)	1:16	1:128	8
GI G106K/L107D (pVAX)	1:8	1:128	16

^a^ These experiments were conducted once

^b^ Under detection limit

To investigate whether the increase in JEV VLPs yield in COS-1 cells under low-temperature cultivation is depended on the positions of amino acid mutations in E protein, we cultured COS-1 cells transfected with plasmids expressing GI and GIII WT and mutated JEV VLPs at either 28 °C or 37 °C for three days. Protein in culture supernatants were collected and measured using Ag-ELISA (Table 1). The results revealed that irrespective of WT or the mutant (W101G, G104H, and G106K/L107D) GI and GIII JEV VLPs, the production was consistently 4–16 times higher under low-temperature conditions at 28 °C compared to cultivation at 37 °C. In summary, these findings suggested that low-temperature cultivation enhances the production of various JEV VLPs in COS-1 cells, regardless of the position of specific amino acid mutations.

### Optimizing low-temperature cultivation conditions

**Fig. 2:**
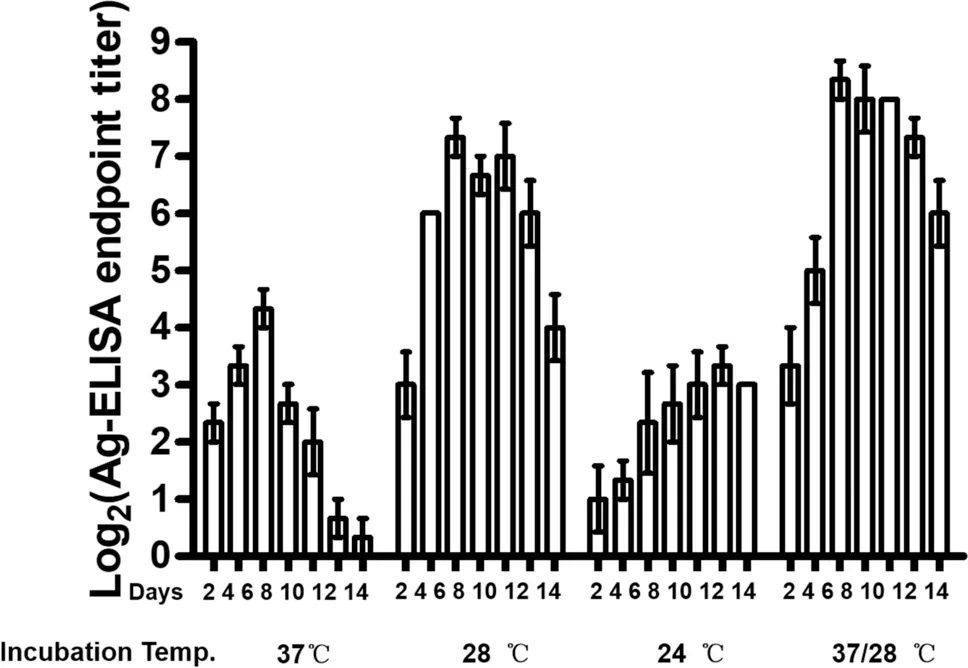
Optimization of the cultivation temperature conditions of plasmid-transfected COS-1 cells to enhance the yield of JEV WT VLPs. The JEV WT VLPs expressing plasmid-transfected COS-1 cells were cultured at 24℃, 28℃, 37℃, or 37/28℃ (cultured at 37℃ for 6 h before shifting to 28℃) for 14 days, culture supernatants collected every 2 days, and VLPs measured by Ag-ELISA. These kinetic experiments were repeated three times.

To determine if 28 °C cultivation represents the optimal condition, COS-1 cells transfected with plasmids expressing WT JEV VLPs were cultured at 24 °C, 28 °C, 37 °C, and 37/28 °C (cultured at 37 °C for 6 h before switching to 28 °C) for 14 days. Culture supernatants were collected every 2 days, and VLP yields were compared using Ag-ELISA (Fig. 2). The results indicated that the Ag-ELISA end-point titer of VLPs at 28 °C was higher than cultured at both 37 °C and 24 °C. The plasmid-transfected cells cultured at 37/28 °C exhibited a gradual increase in VLPs, surpassing the yields obtained at 37 °C and 28 °C switch after day 6, ultimately reaching the highest Ag-ELISA titer of 2^8^. Notably, we observed complete detachment of cells cultured at 37 °C after day 10, while most cells cultured under other conditions remained intact. These results inferred that the optimal condition for producing JEV VLPs in electroporated and plasmid-transfected COS-1 cells involves an initial culture at 37 °C for 6 h followed by a shift to 28 °C for continued cultivation.

### Optimized 37/28 °C cultivation condition increases the production of flavivirus VLPs

To investigate whether optimized 37/28 °C cultivation can enhance the yield of other flavivirus VLPs in COS-1 cell lines, COS-1 cells transfected with plasmids expressing WT GI and GIII JEV, WNV, SLEV, and DENV1-4 VLPs were cultured at either 37/28 °C or 37 °C for six days. The VLPs were then titrated using Ag-ELISA (Table 2). The results demonstrated that the production of all flavivirus VLPs increased significantly under the optimized low-temperature shift from 37 °C to 28 °C, ranging from 5.03 to 20.11 times higher compared to cultivation at 37 °C alone (*P* < 0.05).

**Table 2:** The production of flaviviruses WT VLPs in plasmid-transfected COS-1 cells cultured at 37℃ and 37/28℃

Viral VLP plasmids	Ag-ELISA (log_2_end-point titer)^a^		Fold increased
	37℃	37/28℃	
GIII JEV	5.33(± 0.58^b^)	8.33(± 0.58)	8.00
GI JEV	4.33(± 0.58)	8.66(± 0.58)	20.11
WNV	5.67(± 0.58)	9.00(± 0.00)	10.06
SLEV	1.67(± 0.58)	4.33(± 0.58)	6.32
DENV-1	2.00(± 1.00)	4.33(± 1.00)	5.03
DENV-2	1.67(± 0.58)	5.33(± 0.58)	12.64
DENV-3	4.33(± 0.58)	7.00(± 1.00)	6.36
DENV-4	1.33(± 0.58)	3.66(± 0.58)	5.03

^a^ These experiments were repeated three times

^b^ Standard deviation

Subsequently, to investigate whether optimized 37/28 °C cultivation conditions could enhance the production of flavivirus VLPs in different mammalian cell lines, Vero, CHO, and 293 T cells transfected with plasmids expressing WT JEV GIII, WNV, DENV-1, or DENV-2 VLPs were cultured at either 37 °C or 37/28 °C for four days. Culture supernatants were collected, and VLP yields were compared using Ag-ELISA titration (Table 3). In all cases, the Ag-ELISA end-point titers of samples collected from transfected cells cultured at 37/28 °C were significantly higher, ranging from 5.06 to 20.25 times, compared to cultivation at 37 °C (*P* < 0.05).

**Table 3:** The production of flaviviruses WT VLPs in plasmid-transfected mammal cells cultured at 37℃ and 37/28℃

VLP Plasmids	Ag-ELISA (log_2_end-point titer)^a^								
	Vero cells			CHO-K1 cells			293 T cells		
	37℃	37/28℃	Fold increased	37℃	37/28℃	Fold increased	37℃	37/28℃	Fold increased
GIII JEV	4.33(± 0.58)	6.67(± 0.58)	5.06	4.67(± 0.58)	8.00(± 1.00)	10.06	ND	ND	
WNV	ND^b^	ND		5.33(± 0.58)	9.33(± 0.58)	16.00	ND	ND	
DENV-1	ND	ND		2.33(± 0.58)	4.67(± 0.58)	5.06	2.67(± 0.58)	6.00(± 1.00)	10.06
DENV-2	ND	ND		ND	ND		1.33(± 0.58)	5.67(± 1.00)	20.25

^a^ These experiments were repeated three times

^b^ Not done

Finally, to investigate whether optimized 37/28 °C cultivation conditions could increase the yield of flavivirus VLPs in clonally selected, stably expressing cell lines expressing WT GIII JEV (COS-1), GI JEV (CHO-K1), WNV (COS-1), and DENV-2 (293 T) VLPs were cultured at either 37 °C or 37/28 °C for four days. Subsequently, culture supernatants were collected, and VLP yields were compared using Ag-ELISA titration and absolute quantification (Table 4). In all cases, the Ag-ELISA end-point titers of samples collected from clone cells cultured at 37/28 °C were significantly higher than those at 37 °C (*P* < 0.05), reaching 1889.3, 1910.6, 2507.2, and 974.5 ng/ml for GIII JEV, GI JEV, WNV, and DENV2, respectively.

**Table 4:** The production of flaviviruses WT VLPs in stable clones cultured at 37℃ and 37/28℃^a^

Viral VLP plasmids	Stable cell lines	37℃		37/28℃	
		Ag-ELISA (log_2_end-point titer)	Quantification of E antigen (ng/ml)	Ag-ELISA (log_2_end-point titer)	Quantification of E antigen (ng/ml)
GIII JEV	COS-1	5.67(± 0.58)	ND^b^	8.67(± 0.58)	1889.3(± 121.6)
GI JEV	CHO-K1	5.33(± 0.58)	ND	9.33(± 0.58)	1910.6(± 111.8)
WNV	COS-1	6.00(± 1.00)	ND	10.00(± 0.00)	2507.2(± 455.4)
DENV-2	293 T	2.00(± 0.00)	ND	6.33(± 0.58)	974.5(± 247.1)

^a^ These experiments were repeated three times

^b^ ND: not done

### Antigenic structure of flavivirus VLPs derived from 37/28 °C

**Fig. 3:**
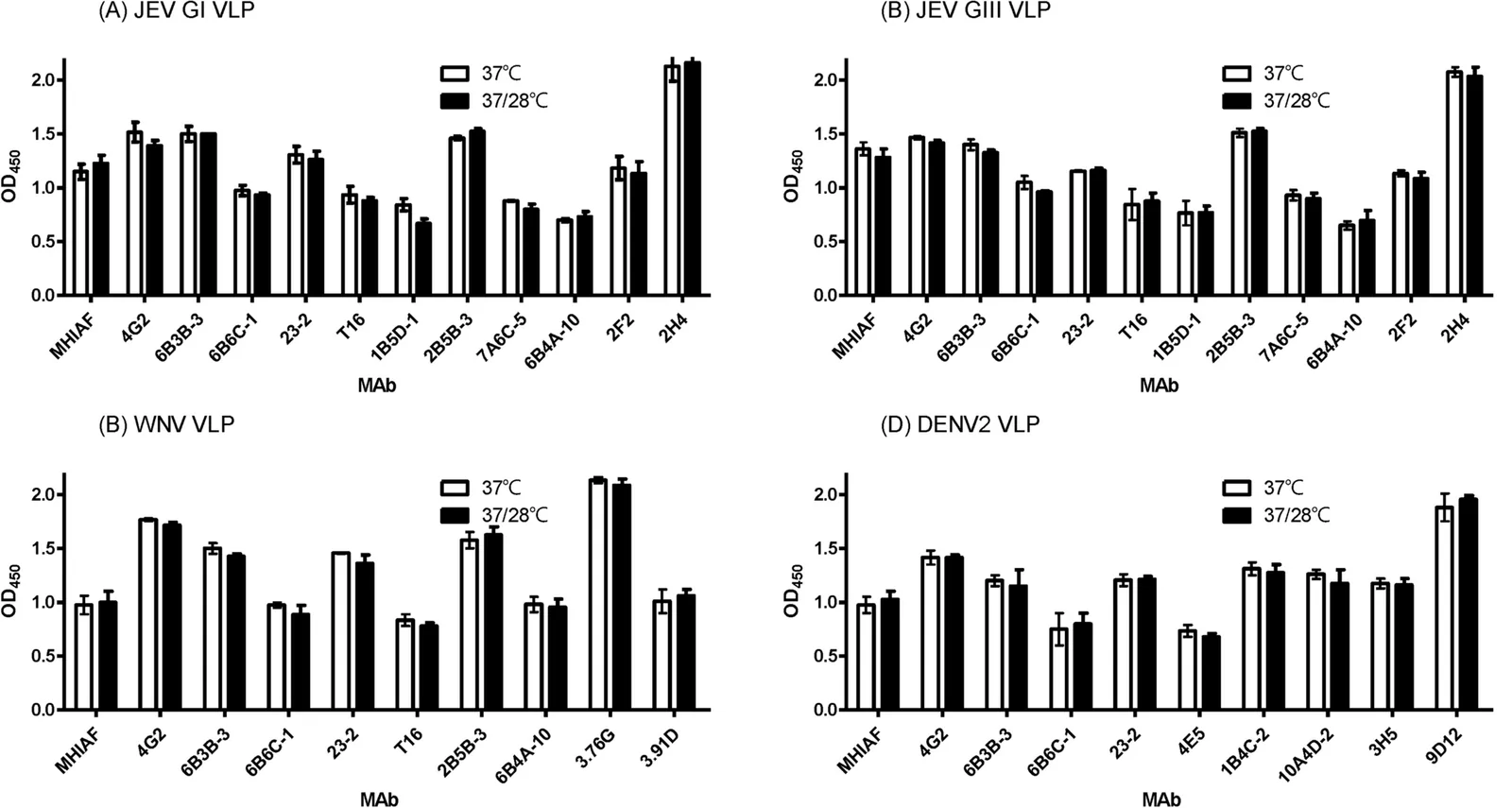
Monoclonal antibodies reactivity of 37℃- and 37/28℃-derived JEV, WNV, and DENV2 WT VLPs. The monoclonal antibodies include group-cross-reactive (4G2, 6B3B-3, 6B6C-1, and 23–2) and complex-cross-reactive (T16, 1B5D-1, 2B5B-3, 7A6C-5, and 6B4A-10 for JEV-serocomplex; 4E5, 1B4C-2, and 10A4D-2 for DENV-serocomplex), and type-specific (2F2 and 2H4 for JEV; 3.76G and 3.91D for WNV; and 3H5 and 9D12 for DENV-2) monoclonal antibodies. The binding capacity between GI JEV (**A**), GIII JEV (**B**), WNV (**C**), and DENV-2 (**D**) WT VLPs and monoclonal antibodies was assessed using an Ag-ELISA method. These experiments were repeated three times.

Previous research has demonstrated that flavivirus VLPs produced under 37 °C cultivation conditions closely resemble the viral particle structure (Konishi et al. 2001). Our previous studies demonstrated that the average diameter of JEV GI and GIII WT VLPs produced at 37℃ or 28℃ ranged from 35 to 40 nm. Both sets of VLPs, composed of E, prM, and M proteins, exhibited indications of partial maturation (Fan et al. 2018; Hunt et al. 2001). To investigate whether the optimized cultivation conditions at 37/28 °C preserve the structural integrity of flavivirus VLPs, we conducted binding affinity analyses of GI JEV, GIII JEV, WNV, and DENV2 VLPs produced at both 37 °C and 37/28 °C cultivation conditions with a panel of monoclonal antibodies (Fig. 3). These monoclonal antibodies included those that were group-cross-reactive (4G2, 6B3B-3, 6B6C-1, and 23–2), complex-cross-reactive (T16, 1B5D-1, 2B5B-3, 7A6C-5, and 6B4A-10 for JEV-serocomplex; 4E5, 1B4C-2, and 10A4D-2 for DENV-serocomplex), and type-specific (2F2 and 2H4 for JEV; 3.76G and 3.91D for WNV; and 3H5 and 9D12 for DENV-2) monoclonal antibodies (Table S1). The binding capacity between VLPs and monoclonal antibodies was assessed using an Ag-ELISA method (Fig. 3). For all VLPs, including JEV GI (Fig. 3A), JEV GIII (Fig. 3B), WNV (Fig. 3C), and DENV-2 (Fig. 3D), prepared at both 37 °C and 37/28 °C, the difference in OD_450_ values for antibody binding did not exhibit statistical significance (P > 0.05). These results suggest that optimizing the low-temperature switch from 37 °C to 28 °C cultivation conditions did not alter the structure of various flavivirus VLPs that were detectable by our panel of monoclonal antibodies.

## Discussion

Inactivated vaccines require chemical or physical methods to inactivate the infectivity of virions, but this process has the potential to damage viral proteins or alter their antigenic structures, affecting the induction of protective immunity (Wilton et al. 2014). For instance, the formalin-inactivated JEV vaccine disrupts critical epitopes in domain III of the E protein responsible for inducing neutralizing antibodies (Fan et al. 2015). In contrast, licensed HBV and HPV VLPs vaccines have demonstrated outstanding efficacy, because their structures closely resemble virus particles, eliminating the need for inactivation, and allowing for the induction of highly effective protective immunity (Zhao et al. 2013). Our previous report demonstrated that no virus or viral RNA could be detected in the blood or other tissues of pigs immunized with GI JEV VLPs following viral challenge, and suggested the potential of this vaccine to induce highly desirable and effective immunity (Fan et al. 2018). Other literature also indicates favorable results in animal testing for several flavivirus VLPs vaccines, including ZIKV and DENV (Boigard et al. 2018; Garg et al. 2020; Urakami et al. 2017).

In many studies of flavivirus DNA vaccines, it has been observed that optimal protection is achieved when translated virus proteins can be efficiently released into the extracellular environment (Chang et al. 2000). This observation has sparked interest in developing VLPs vaccines. To obtain flavivirus VLPs, researchers have undertaken to optimize the expressing DNA plasmid, including enhancing the efficiency of transcription and translation within cells (Chang et al. 2000), ensuring the correct intracellular localization of virus prM and E proteins by incorporating capsid protein signal sequences (Chang et al. 2000), and facilitating the assembly and budding of VLPs by optimizing the E protein transmembrane domain sequence (Purdy and Chang 2005). Although these optimizations successfully yield extracellular particles or VLPs, some flavivirus VLPs, such as DENV-2 and -4, still exhibit limited production yields (Table 2).

A previous report indicated that clonally selected, stable cell lines continuously expressing JEV VLPs produced yields ranging from 34 to 270 ng/ml under conventional 37 °C cultivation conditions (Konishi et al. 2001). However, in our current study, stable cell lines expressing flavivirus VLPs consistently achieved yields exceeding 1 μg/ml when cultivated at 37/28 °C (Table 4). Cultivation in a serum-free medium yielded similar results (data not shown), and cell culture supernatants could be collected for over 14 days (Fig. 2). Immunization of pigs with 2 doses of 5 μg GI JEV VLPs triggered highly effective immunity. We concluded that the developed culture conditions yield sufficient flavivirus VLPs to meet the requirements for vaccine production.

The alphavirus SFV with mutations G91D or G91A in the E1 protein fusion peptide reduced the production of virus particles in BHK-21 cells (Duffus et al. 1995). However, if infected cells are cultured at 28 °C, virus production can be effectively restored. When conducting amino acid mutation experiments in the E protein fusion peptide using flavivirus VLPs expression plasmids, they observed that mutations at positions 104 and 107 led to a substantial reduction in VLPs production (Chiou et al. 2008; Crill et al. 2007; Roberson et al. 2007), similar to the situation observed in the context of SFV. Following the approach used for SFV, the production of JEV VLPs with the G104H mutation was successfully restored (Chiou et al. 2008). Furthermore, this study demonstrated that the increase in VLPs production at low-temperature cultivation was not dependent on amino acid mutations (Table 1), mammalian cell types (Tables 3 and 4), or flavivirus species (Table 2).

Mutations G91D or G91A in the SFV E1 protein fusion peptide have been observed to reduce the stability of the E1-E2 protein heterodimer during the late stages of virus assembly (Duffus et al. 1995). Cultivating infected cells at 28 °C could potentially prolong the time available for viral protein assembly on the cell membrane, facilitating the formation of E1 and E2 protein dimers and ultimately restoring the production of virus particles. The prM protein stabilizes the JEV E protein fusion peptide to prevent conformational rearrangement in the acidic compartment during the exocytosis process. Since the expression of viral proteins did not appear to be affected (Fig. 1B), we speculated that culturing cells transiently expressing flavivirus VLPs at 37/28 °C may increase the stability of the prM-E protein interaction, thereby effectively restoring and enhancing VLPs production. Further research is needed to determine its possible mechanism(s).

The results of cryoelectron microscopy analysis revealed differences in the structure of DENV at different temperatures, with a bumpy surface observed at 37 °C and a smoother surface at 28 °C (Zhang et al. 2013). Additionally, WNV may undergo a conformational transition between 37 °C and 43 °C (Kaufmann et al. 2006, 2010). The variation in the temperature threshold for conformational transition between DENV and WNV may be associated with the hosts in their transmission cycle, such as humans (37 °C) for DENV and birds (43 °C) for WNV. In this study, through a comprehensive analysis using MAbs mapping analysis, the antigenic structure of VLPs produced from cultivation at 37 °C or 37/28 °C appeared to be similar with all VLP samples stored at 4 °C before analysis. Further investigation is required to determine the impact of the bumpy or smooth surface of the virus/VLPs on vaccine design, manufacturing, and storage.

## Supplementary Information

Below is the link to the electronic supplementary material.

Supplementary file1 (PDF 511 KB)

## Data Availability

The publication contains all the supporting information for the study’s findings, and the corresponding author is also willing to provide it upon reasonable request.
