# Supplementary material for: Low-temperature culture enhances production of flavivirus virus-like particles in mammalian cells
Source: Appl Microbiol Biotechnol. 2024 Feb 28;108:242. doi: 10.1007/s00253-024-13064-y (PMC10902078; doi:10.1007/s00253-024-13064-y)
Supplement: Supplementary file 1 [file 253_2024_13064_MOESM1_ESM.pdf]

*Applied Microbiology and Biotechnology*

# Low-temperature culture enhances production of flavivirus virus-like particles in mammalian cells

Yi-Chin Fan [1,2], Jo-Mei Chen [1], Yi-Ying Chen [1], Wei-Li Hsu [1], Gwong-Jen Chang [3], and Shyan-Song Chiou [1]

[1] Graduate Institute of Microbiology and Public Health, National Chung Hsing University, Taichung 402, Taiwan

[2] Institute of Epidemiology and Preventive Medicine, College of Public Health, National Taiwan University, Taipei 10617, Taiwan

[3] Arboviral Diseases Branch, Centers for Disease Control and Prevention, Fort Collins, Colorado 80521, United States of America

Addresses of authors:

YCF: 17, Xu-Zhou Road, Taipei 10617, Taiwan;

JMC, YYC, WLH, and SSC: 145 Xingda Rd., South Dist., Taichung City 402, Taiwan;

GJC: 3156 Rampart Road, Fort Collins, CO 80521, United States

Corresponding author: Shyan-Song Chiou
E-mail: sschiou@dragon.nchu.edu.tw;
Telephone: +886-4-22840695;
Fax: +886-4-22851741.

**Supplementary information**

**Table S1. Polyclonal and monoclonal antibodies used for VLPs antigenic structural characterization in this study**

| Antibody type | Species | MHIAF or MAb | Immunogen | Reactivity | Reference | Used in this study |
|---|---|---|---|---|---|---|
| Polyclonal | | | | | | |
| | Rabbit | Anti-JEV MHIAF | JEV prM/E DNA | | (Chiou et al. 2008) | Ag-ELISA as capture Ab |
| | Rabbit | Anti-WNV MHIAF | WNV prM/E DNA | | (Crill et al. 2007) | Ag-ELISA as capture Ab |
| | Rabbit | Anti-SLEV MHIAF | SLEV prM/E DNA | | (Trainor et al. 2007) | Ag-ELISA as capture Ab |
| | Rabbit | Anti-DENV-1 MHIAF | DENV-1 prM/E DNA | | (Purdy and Chang 2005) | Ag-ELISA as capture Ab |
| | Rabbit | Anti-DENV-2 MHIAF | DENV-2 prM/E DNA | | (Purdy and Chang 2005) | Ag-ELISA as capture Ab |
| | Rabbit | Anti-DENV-3 MHIAF | DENV-3 prM/E DNA | | (Purdy and Chang 2005) | Ag-ELISA as capture Ab |
| | Rabbit | Anti-DENV-4 MHIAF | DENV-4 prM/E DNA | | (Purdy and Chang 2005) | Ag-ELISA as capture Ab |

| | | | | | | |
|---|---|---|---|---|---|---|
| | Mouse | Anti-JEV MHIAF | JEV | | (Chiou et al. 2008) | Ag-ELISA as detector Ab |
| | Mouse | Anti-WNV MHIAF | WNV | | (Crill et al. 2007) | Ag-ELISA as detector Ab |
| | Mouse | Anti-SLEV MHIAF | SLEV | | (Trainor et al. 2007) | Ag-ELISA as detector Ab |
| | Mouse | Anti-DENV-1 MHIAF | DENV-1 | | (Purdy and Chang 2005) | Ag-ELISA as detector Ab |
| | Mouse | Anti-DENV-2 MHIAF | DENV-2 | | (Purdy and Chang 2005) | Ag-ELISA as detector Ab |
| | Mouse | Anti-DENV-3 MHIAF | DENV-3 | | (Purdy and Chang 2005) | Ag-ELISA as detector Ab |
| | Mouse | Anti-DENV-4 MHIAF | DENV-4 | | (Purdy and Chang 2005) | Ag-ELISA as detector Ab |
| Monoclonal | | | | | | |
| | Mouse | 4G2 | DENV-2 | GCR[a] | (Chiou et al. 2008) | VLPs characterization |
| | Mouse | 6B3B-3 | SLEV | GCR | (Chiou et al. 2008) | VLPs characterization |
| | Mouse | 6B6C-1 | SLEV | GCR | (Chiou et al. 2008) | VLPs characterization |
| | Mouse | 23-2 | JEV | GCR | (Chiou et al. | VLPs characterization |

| | | | | | | |
|---|---|---|---|---|---|---|
| | | | | | 2008) | |
| | Mouse | T16 | JEV | JEV-CCR[b] | (Chiou et al. 2008) | VLPs characterization |
| | Mouse | 1B5D-1 | SLEV | JEV-CCR | (Chiou et al. 2008) | VLPs characterization |
| | Mouse | 2B5B-3 | SLEV | JEV-CCR | (Chiou et al. 2008) | VLPs characterization |
| | Mouse | 7A6C-5 | unknown | JEV-CCR | (Chiou et al. 2008) | VLPs characterization |
| | Mouse | 6B4A-10 | JEV | JEV-CCR | (Chiou et al. 2008) | VLPs characterization |
| | Mouse | 4E5 | DENV-2 | DENV-CCR | (Crill and Chang 2004) | VLPs characterization |
| | Mouse | 1B4C-2 | DENV-2 | DENV-CCR | (Crill and Chang 2004) | VLPs characterization |
| | Mouse | 10A4D-2 | DENV-2 | DENV-CCR | (Crill and Chang 2004) | VLPs characterization |
| | Mouse | 2F2 | JEV | JEV-specific | (Chiou et al. 2008) | VLPs characterization |
| | Mouse | 2H4 | JEV | JEV-specific | (Chiou et al. 2008) | VLPs characterization |
| | Mouse | 3.76G | WNV | WNV-specific | (Crill et al. | VLPs characterization |

| | | | | | | |
|---|---|---|---|---|---|---|
| | | | | | 2007) | |
| | Mouse | 3.91D | WNV | WNV-specific | (Crill et al. 2007) | VLPs characterization |
| | Mouse | 3H5 | DENV-2 | DENV-2-specific | (Crill and Chang 2004) | VLPs characterization |
| | Mouse | 9D12 | DENV-2 | DENV-2-specific | (Crill and Chang 2004) | VLPs characterization |

[a] GCR: group cross-reactive MAb

[b] CCR: complex cross-reactive MAb
