# Supplementary material for: Low-temperature culture enhances production of flavivirus virus-like particles in mammalian cells
Source: Appl Microbiol Biotechnol. 2024 Feb 28;108(1):242. doi: 10.1007/s00253-024-13064-y (PMC10902078; doi:10.1007/s00253-024-13064-y)
Supplement: Supplementary file 1 — Supplementary file1 (PDF 511 KB) [file 253_2024_13064_MOESM1_ESM.pdf]

Applied Microbiology and Biotechnology

Low-temperature culture enhances production of flavivirus virus-like particles in mammalian cells

Yi-Chin Fan [1,2], Jo-Mei Chen [1], Yi-Ying Chen [1], Wei-Li Hsu [1], Gwong-Jen Chang [3], and Shyan-Song Chiou [1]

[1] Graduate Institute of Microbiology and Public Health, National Chung Hsing University, Taichung 402, Taiwan

[2] Institute of Epidemiology and Preventive Medicine, College of Public Health, National Taiwan University, Taipei 10617, Taiwan

[3] Arboviral Diseases Branch, Centers for Disease Control and Prevention, Fort Collins, Colorado 80521, United States of America

Addresses of authors:

YCF: 17, Xu-Zhou Road, Taipei 10617, Taiwan;

JMC, YYC, WLH, and SSC: 145 Xingda Rd., South Dist., Taichung City 402, Taiwan;

GJC: 3156 Rampart Road, Fort Collins, CO 80521, United States

Corresponding author: Shyan-Song Chiou
E-mail: sschiou@dragon.nchu.edu.tw;
Telephone: +886-4-22840695;
Fax: +886-4-22851741.

Supplementary information

Table S1. Polyclonal and monoclonal antibodies used for VLPs antigenic structural characterization in this study

Antibody type	Species	MHIAF or MAb	Immunogen	Reactivity	Reference	Used in this study
Polyclonal						
	Rabbit	Anti-JEV MHIAF	JEV prM/E DNA		(Chiou et al. 2008)	Ag-ELISA as capture Ab
	Rabbit	Anti-WNV MHIAF	WNV prM/E DNA		(Crill et al. 2007)	Ag-ELISA as capture Ab
	Rabbit	Anti-SLEV MHIAF	SLEV prM/E DNA		(Trainor et al. 2007)	Ag-ELISA as capture Ab
	Rabbit	Anti-DENV-1 MHIAF	DENV-1 prM/E DNA		(Purdy and Chang 2005)	Ag-ELISA as capture Ab
	Rabbit	Anti-DENV-2 MHIAF	DENV-2 prM/E DNA		(Purdy and Chang 2005)	Ag-ELISA as capture Ab
	Rabbit	Anti-DENV-3 MHIAF	DENV-3 prM/E DNA		(Purdy and Chang 2005)	Ag-ELISA as capture Ab
	Rabbit	Anti-DENV-4 MHIAF	DENV-4 prM/E DNA		(Purdy and Chang 2005)	Ag-ELISA as capture Ab

	Mouse	Anti-JEV MHIAF	JEV		(Chiou et al. 2008)	Ag-ELISA as detector Ab
	Mouse	Anti-WNV MHIAF	WNV		(Crill et al. 2007)	Ag-ELISA as detector Ab
	Mouse	Anti-SLEV MHIAF	SLEV		(Trainor et al. 2007)	Ag-ELISA as detector Ab
	Mouse	Anti-DENV-1 MHIAF	DENV-1		(Purdy and Chang 2005)	Ag-ELISA as detector Ab
	Mouse	Anti-DENV-2 MHIAF	DENV-2		(Purdy and Chang 2005)	Ag-ELISA as detector Ab
	Mouse	Anti-DENV-3 MHIAF	DENV-3		(Purdy and Chang 2005)	Ag-ELISA as detector Ab
	Mouse	Anti-DENV-4 MHIAF	DENV-4		(Purdy and Chang 2005)	Ag-ELISA as detector Ab
Monoclonal						
	Mouse	4G2	DENV-2	GCR[a]	(Chiou et al. 2008)	VLPs characterization
	Mouse	6B3B-3	SLEV	GCR	(Chiou et al. 2008)	VLPs characterization
	Mouse	6B6C-1	SLEV	GCR	(Chiou et al. 2008)	VLPs characterization
	Mouse	23-2	JEV	GCR	(Chiou et al.	VLPs characterization

					2008)	
	Mouse	T16	JEV	JEV-CCR[b]	(Chiou et al. 2008)	VLPs characterization
	Mouse	1B5D-1	SLEV	JEV-CCR	(Chiou et al. 2008)	VLPs characterization
	Mouse	2B5B-3	SLEV	JEV-CCR	(Chiou et al. 2008)	VLPs characterization
	Mouse	7A6C-5	unknown	JEV-CCR	(Chiou et al. 2008)	VLPs characterization
	Mouse	6B4A-10	JEV	JEV-CCR	(Chiou et al. 2008)	VLPs characterization
	Mouse	4E5	DENV-2	DENV-CCR	(Crill and Chang 2004)	VLPs characterization
	Mouse	1B4C-2	DENV-2	DENV-CCR	(Crill and Chang 2004)	VLPs characterization
	Mouse	10A4D-2	DENV-2	DENV-CCR	(Crill and Chang 2004)	VLPs characterization
	Mouse	2F2	JEV	JEV-specific	(Chiou et al. 2008)	VLPs characterization
	Mouse	2H4	JEV	JEV-specific	(Chiou et al. 2008)	VLPs characterization
	Mouse	3.76G	WNV	WNV-specific	(Crill et al.	VLPs characterization

					2007)	
	Mouse	3.91D	WNV	WNV-specific	(Crill et al. 2007)	VLPs characterization
	Mouse	3H5	DENV-2	DENV-2-specific	(Crill and Chang 2004)	VLPs characterization
	Mouse	9D12	DENV-2	DENV-2-specific	(Crill and Chang 2004)	VLPs characterization

[a] GCR: group cross-reactive MAb

[b] CCR: complex cross-reactive MAb
